# Characteristic Features of Bimaxillary Proclination: A 5-Year Review in Najran, a Saudi Arabian Sub-population

**DOI:** 10.7759/cureus.68893

**Published:** 2024-09-07

**Authors:** Bandar Alyami

**Affiliations:** 1 Preventive Dentistry Department, College of Dentistry, Najran University, Najran, SAU

**Keywords:** bi-dental protrusion, bimaxillary proclination, lip incompetence, malocclusion, orthodontic

## Abstract

Background

Bimaxillary proclination (BP) has been described as a clinical condition depicted by proclined upper and lower incisors with an amplified lip incompetence. The current study aimed to investigate the prevalence and characteristic features of bimaxillary proclination in Najran.

Methods

This was a retrospective study to appraise bimaxillary proclination in an orthodontic specialist clinic and research complex in Najran, Saudi Arabia from January 2018 to December 2022. Patients requesting orthodontic treatment for malocclusion were evaluated. Data retrieved include: age, gender, presence of bimaxillary proclination, and facial profile of affected patients. IBM SPSS Statistics for IOS Version 25 (Armonk, NY: IBM Corp) was used for analysis. Results were presented as simple frequencies and descriptive statistics.

Results

Three hundred and twenty-six (326) patients sought treatment for different types of malocclusion out of which 277 (84.9%) had bimaxillary proclination. There were 123 (44.4%) males and 154 (55.6%) females with an M:F ratio of 1:1.3. Age ranged from 6 to 55 years with a mean SD of 22.6 ± 8.98. More females had bimaxillary proclination. The majority of the patients with bimaxillary proclination had convex facial profiles and a Class I relationship (241 (73.9%) and (135 (48.7%)), respectively.

Conclusion

Bimaxillary proclination is a common orthodontic health issue with high prevalence in the Najran region. Female preponderance was observed. Most of the patients have a Class I Angle’s relationship.

## Introduction

Bimaxillary proclination (BP) has been described as a clinical condition depicted by proclined upper and lower incisors with an amplified lip incompetence [1]. BP, also referred to as dentoalveolar protrusion or bi-alveolar protrusion, develops when the mandibular and maxillary anterior teeth procline in comparison with cranial and dental bases resulting in a soft tissue prominence [2, 3]. Although the condition is seen in all ethnic groups, it is observed more commonly in the Asian and African-American populations [1, 4]. No specific etiology has been ascribed to the development of BP. However, multifactorial causes such as hereditary and environmental influences comprising mouth breathing, tongue volume, and tongue and lip habits, have been reported as possible etiology of BP [4-7].

Patients with this condition usually have a very poor quality of life (QoL), and therefore, usually seek orthodontic help to correct the anomaly [8-10]. With the increase in health-seeking behavior of patients with bimaxillary proclination, there is a need to have accurate data in every society on the prevalence of bimaxillary proclination. This initiative is to have informed data to guide policy formulators in the allocation of both manpower and material resources to correct the abnormality. The current study, therefore, hopes to review bimaxillary proclination in a referral health institution in Najran, Saudi Arabia as no previous report exists.

## Materials and methods

This was a retrospective study conducted with a convenience sampling method at an orthodontic specialist clinic (Dalma Medical Center) in Najran, Saudi Arabia from January 2018 to December 2022 to determine the prevalence of bimaxillary proclination among orthodontic patients seeking treatment for their malocclusion(s). The study followed the Declaration of Helsinki on the conduct of human research. Ethical approval was not required as the study was retrospective in design. The study population comprises subjects in the age range of 6-55 years requiring orthodontic treatment during the study period. Inclusion criteria were all patients with bi-maxillary proclination with complete data. Diagnosis of bi-maxillary proclination was made by consultant orthodontists using both clinical and radiographic evaluations. Excluded were other cases of malocclusion that were not bi-maxillary proclination. Data collected included socio-demographic details such as age, gender, and region they came from. Furthermore, the presence of bimaxillary proclination and the facial profiles of the patients were also collected.

The required sample size was determined by using the prevalence of 20.0% from a similar study on bimaxillary proclination [11] and a formula for a prevalence study [12] (n= (1.96)2P (100-P)/d2) applied with a confidence level present at 95%. This formula gave a minimum sample size of 246 cases. However, all diagnosed cases of bimaxillary proclination were included.

Data was stored and analyzed using IBM SPSS Statistics for IOS Version 25 (Armonk, NY: IBM Corp). Descriptive statistics were carried out for socio-demographic variables such as age, gender, and the presence or absence of bimaxillary proclination. The descriptive variables that are continuous parameters, such as mean, median, minimum, maximum, and the measures of variability were determined. For descriptive variables that are categorical, simple frequency and percentages were determined. level of significance among categorical variables such as patient’s age group, gender, and the presence or absence of bimaxillary proclination was assessed using Pearson’s chi-square with p ≤ 0.05 considered significant.

## Results

Three hundred and twenty-six (326) patients with different types of malocclusion were appraised out of which 277 (84.9%) had bimaxillary proclination. There were 123 (44.4%) males and 154 (55.6%) females with an M:F ratio of 1:1.3. Age ranged from 6 to 55 years with a mean SD of 22.6 ± 8.98. The majority of the patients are within the age bracket of 11-30 years (Table 1).

**Table 1:** Distribution of age-group of patients according to gender.

	Gender		
Age-group (years)	Male (%)	Female (%)	Total (%)
1-10	9 (2.8)	8 (2.5)	17 (5.2)
11-20	54 (16.6)	84 (25.8)	138 (42.3)
21-30	55 (16.9)	56 (17.2)	111 (34.0)
31-40	16 (4.9)	30 (9.2)	46 (14.1)
41-50	8 (2.5)	5 (1.5)	13 (4.0)
51-60	1 (0.3)	0 (0.0)	1 (0.3)
Total	143 (43.8)	183 (56.2)	326 (100.0)

**Figure 1:**
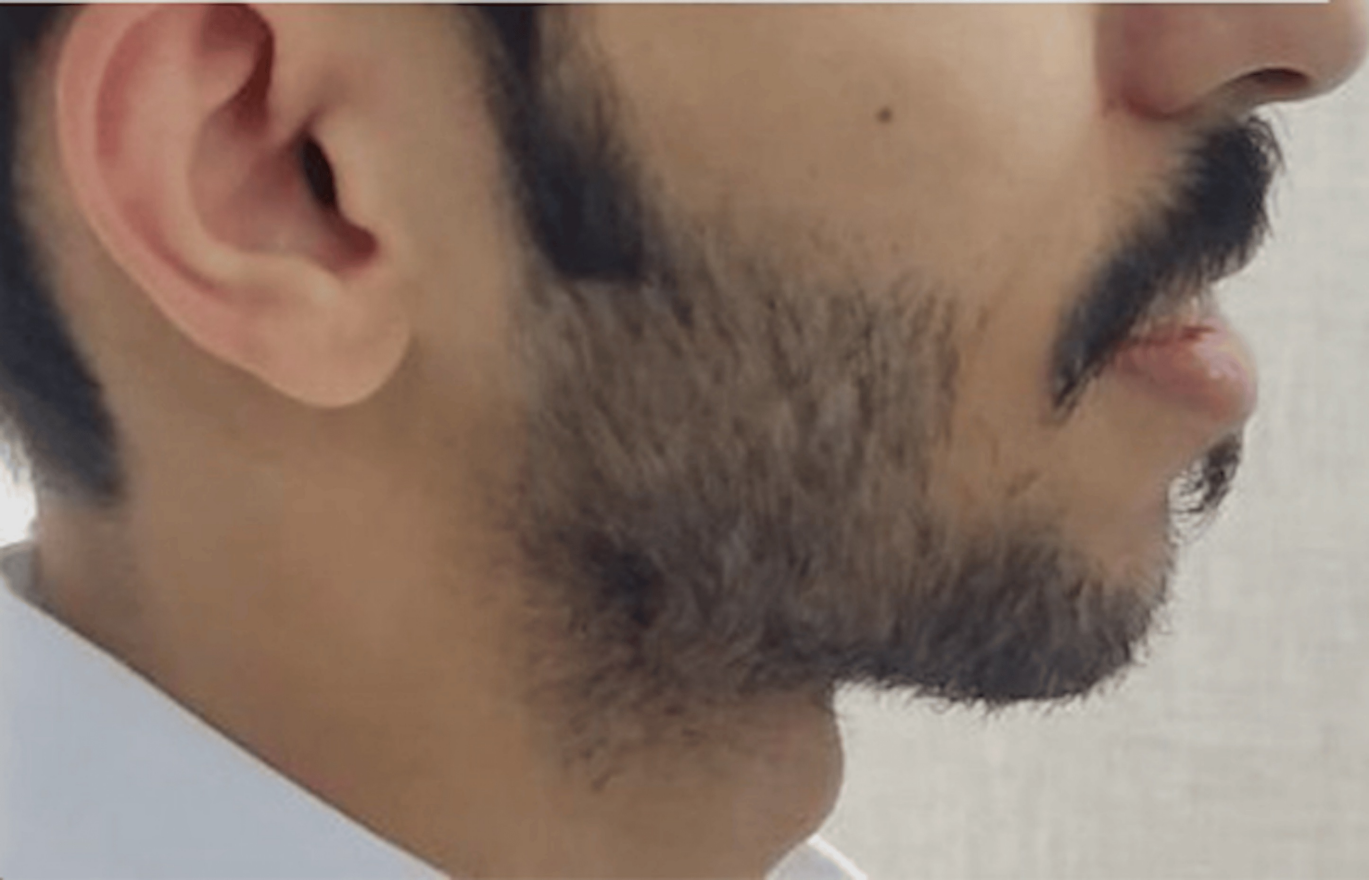
Facial profile of a male patient with bimaxillary proclination.

**Figure 2:**
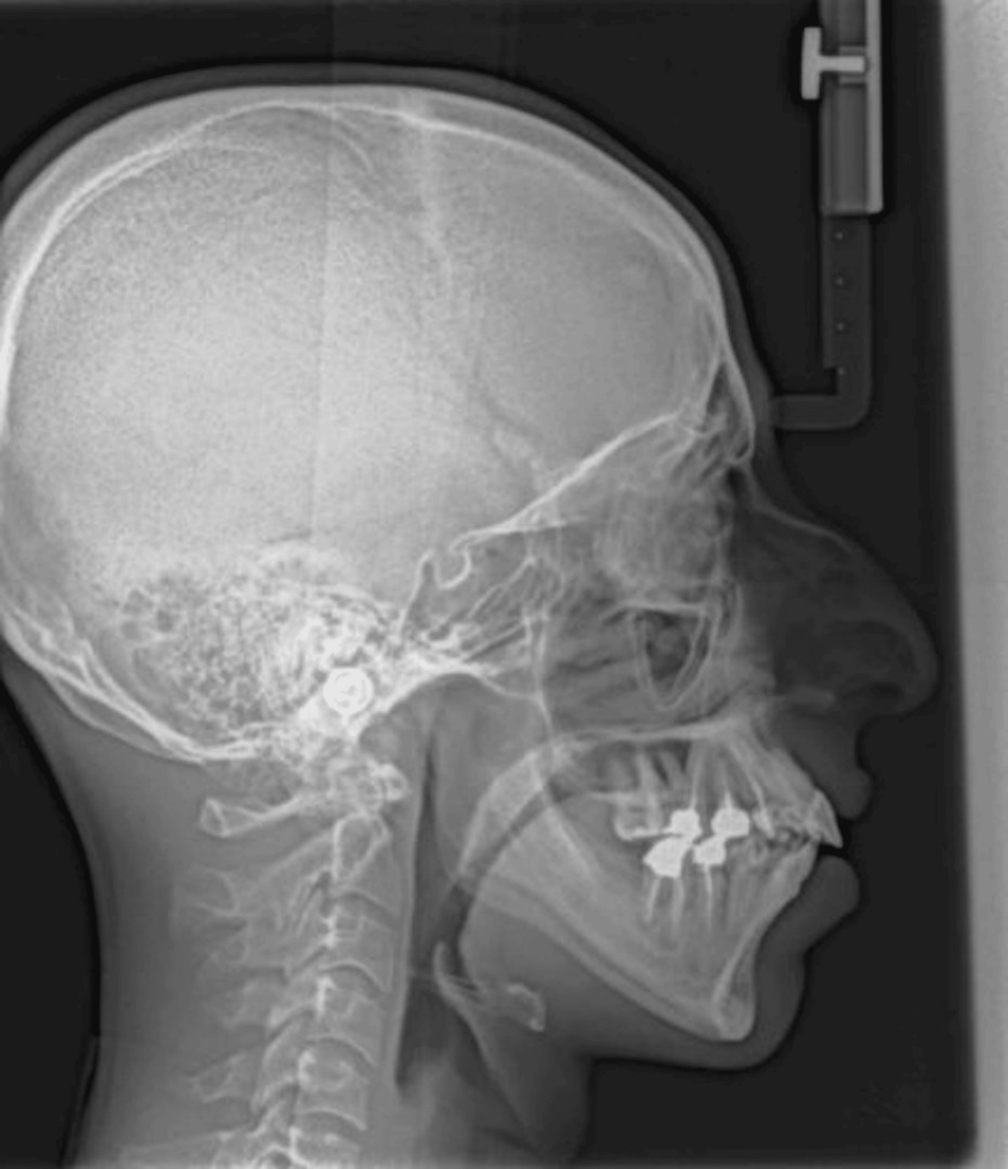
Cephalometry radiograph of male patient in Figure 1 with bimaxillary proclination.

**Figure 3:**
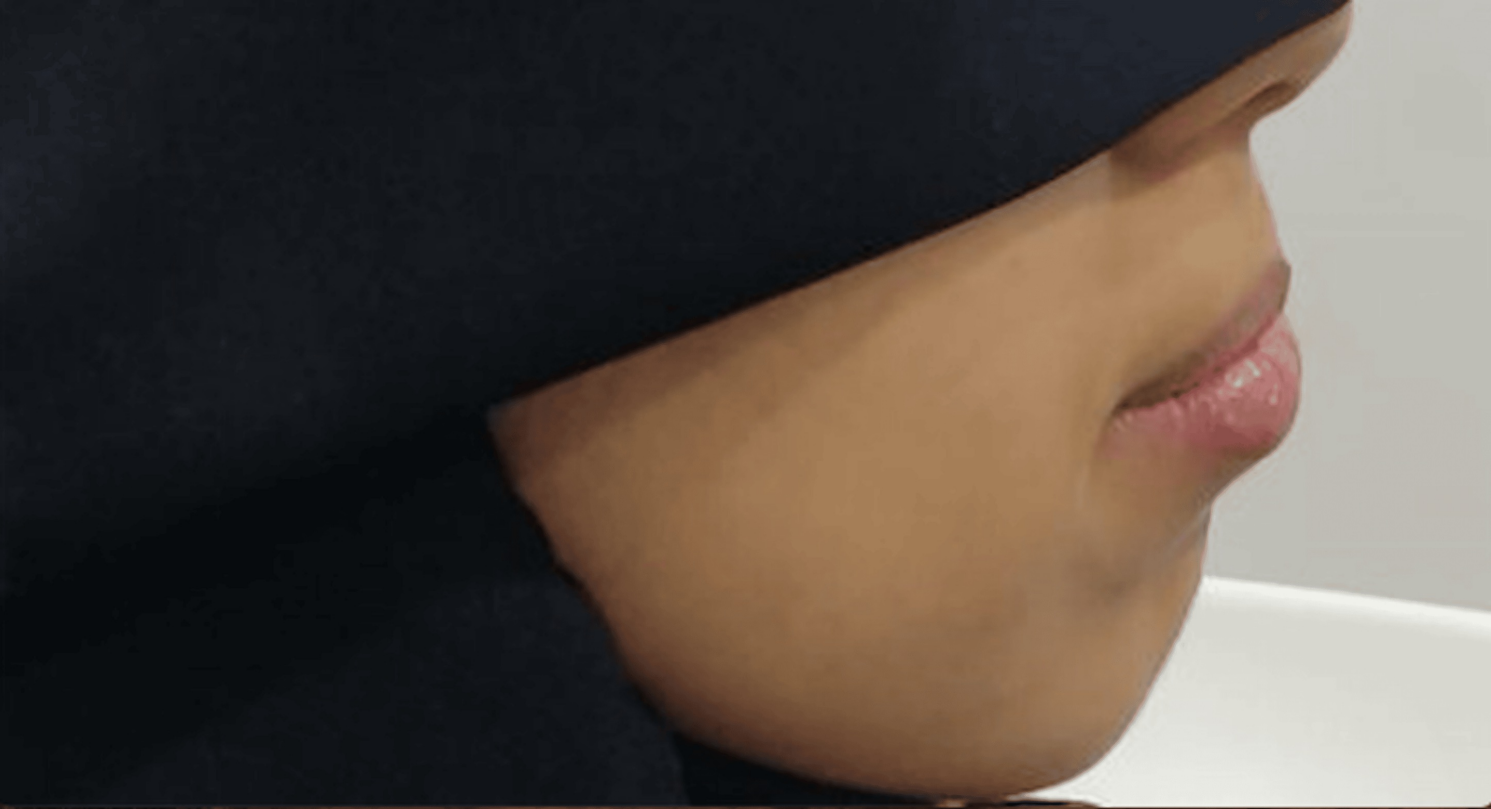
Facial profile of a female patient with bimaxillary proclination.

**Figure 4:**
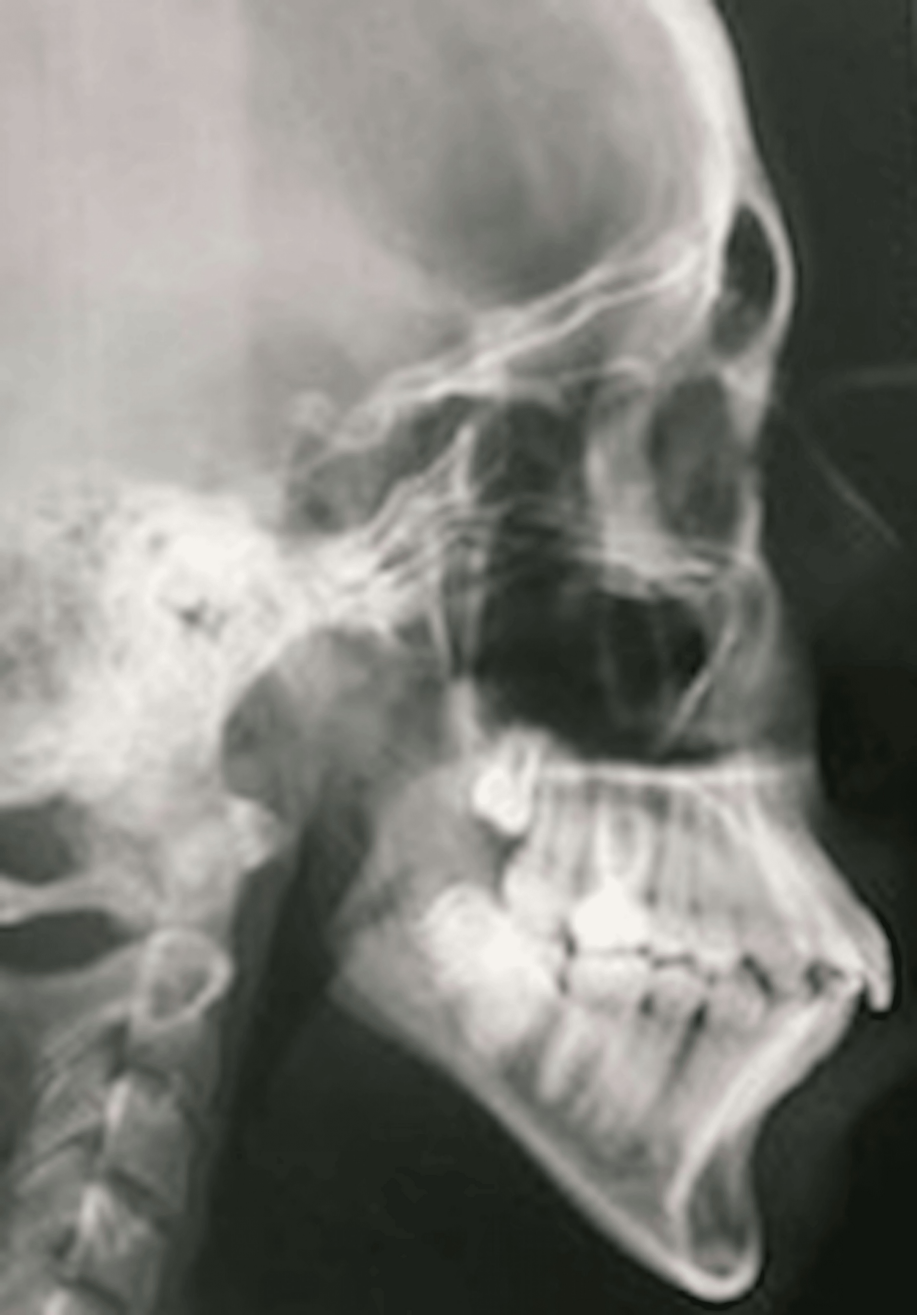
Cephalometry radiograph of female patient in Figure 3 with bimaxillary proclination.

**Figure 5:**
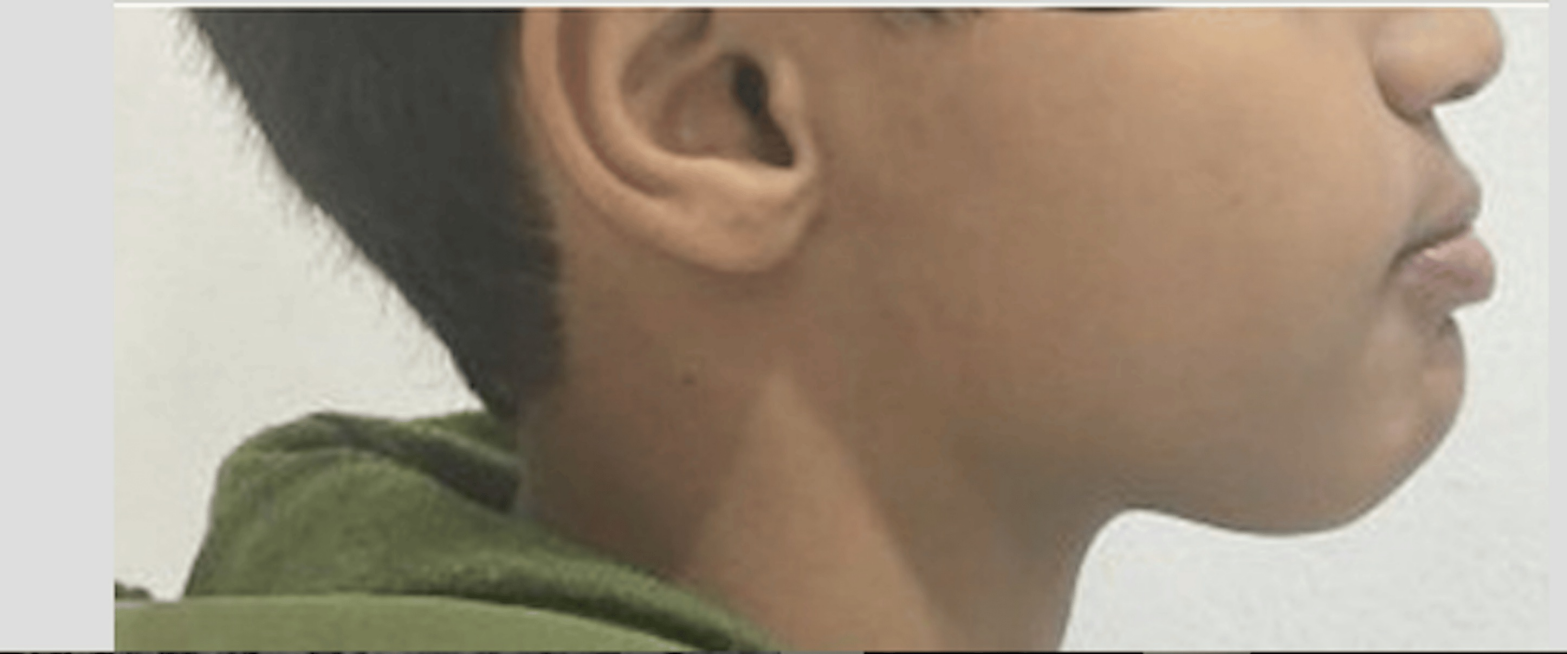
Facial profile of a male child patient with bimaxillary proclination.

**Figure 6:**
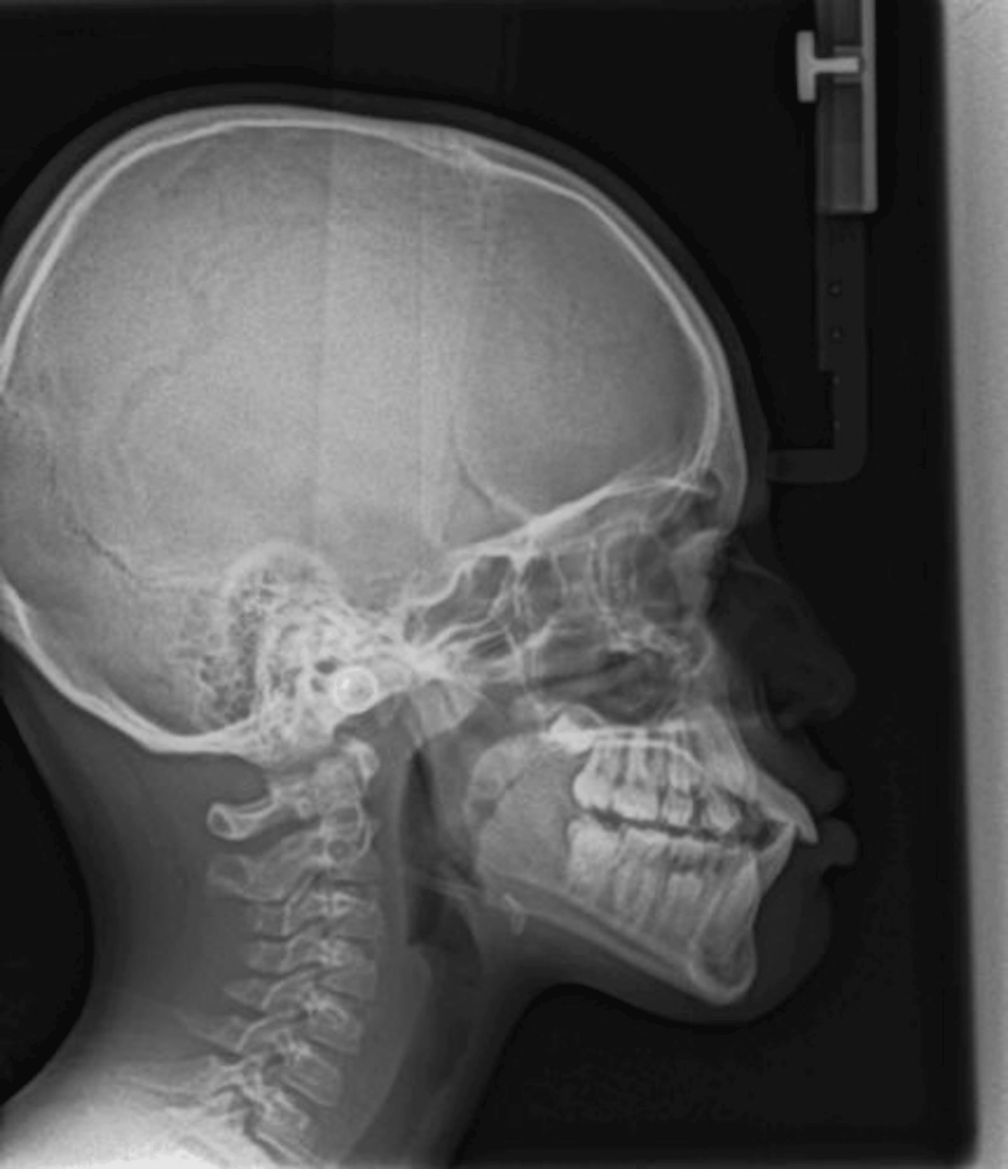
Cephalometry radiograph of male child patient in Figure 5 with bimaxillary proclination.

Figures 1, 2, Figures 3, 4, and Figures 5, 6 show a male, a female, and a child with bimaxillary proclination and their cephalometric radiographs, respectively.

Regarding the presence or absence of bimaxillary proclination according to the gender of patients, more females had bimaxillary proclination, although this was not significant statistically (χ2 =0.218, df=1, p value=0.641) (Table 2).

**Table 2:** Distribution of bi-maxillary proclination and facial profile according to gender

	Gender			
	Male (%)	Female (%)	Total (%)	Statistics
Bi-maxillary proclination				χ2 =0.218, df=1, p value=0.641
Present	123 (37.7)	154 (47.2)	277 (84.9)	
Absent	20 (6.1)	29 (8.9)	49 (15.0)	
Total	143 (43.9)	183 (56.1)	326 (100.0)	
Facial profile				χ2 =0.686, df=2, p value=0.710
Straight	14 (4.3)	17 (5.2)	31 (9.5)	
Convex	120 (36.8)	150 (46.0)	270 (82.8)	
Concave	9 (2.8)	16 (4.9)	25 (7.7)	
Total	143 (43.9)	183 (56.1)	326 (100.0)	

Additionally, the majority of the patients with bimaxillary proclination had convex facial profiles (241 (73.9%)), which was statistically significant χ2 =36.392, df=2, p value=0.000 (Table 3).

**Table 3:** Distribution of facial profile according to bi-maxillary proclination

	Bi-maxillary proclination		
Facial profile	Present (%)	Absent (%)	Total (%)
Straight	15 (4.6)	16 (4.9)	31 (9.5)
Convex	241 (73.9)	29 (8.9)	270 (82.8)
Concave	21 (6.4)	4 (1.2)	25 (7.7)
Total	277 (84.9)	49 (15.0)	326 (100.0)

Concerning the relationship of the bimaxillary proclination with the skeletal base, the majority (135 (48.7%) of the 277 patients had Class I, followed by Class II with 127 (45.8%) patients, and the least was Class III with 15 (5.4%) patients as shown in Table 4.

**Table 4:** Mean distribution of cephalometric evaluation of patients with bimaxillary proclination according to skeletal and incisal relationships SNA: Sella, Nasion, A point; SNB: Sella, Nasion, B point; ANB: A point, Nasion, B point; U1-SN: Upper Incisor-Sella Nasion; L1-NB: Lower Incisor-Nasion B point

	Skeletal base			
	Class I (n=135)	Class II (n=127)	Class III (n=15)	Significance
Skeletal relationship (normal values)	Mean±SD	Mean±SD	Mean±SD	
SNA (82)	81.5 (3.2)	81.7 (3.2)	79.2 (3.2)	NS
SNB (80)	79.8 (2.9)	77.4 (2.9)	81.5 (2.9)	NS
ANB (2)	1.7 (1.1)	4.3 (1.1)	-2.3 (1.1)	NS
Incisal relationship (normal values)	(n=277)	(n=277)	(n=277)	
U1-SN (^0^) (103)	110.2 (3.5)	110.2 (3.5)	110.2 (3.5)	NS
L1-NB (^0^) (25)	34.5 (2.4)	34.5 (2.4)	34.5 (2.4)	NS

## Discussion

Bimaxillary protrusion is usually diagnosed clinically from the facial appearance that is based on lip incompetence, lip strain, and prominent lip in the profile view [13]. This condition can be diagnosed from the mixed dentition stage [14]. The terminology ‘bimaxillary protrusion’ is synonymous with bi-alveolar protrusion, bi-maxillary dental protrusion, bi-maxillary prognathism, bi-maxillary dentoalveolar protrusion, and bi-dental protrusion [15]. The prevalence of bimaxillary proclination has been reported to vary globally ranging from 3,7% to 68% [16-18]. This extensive range might be due to geographic location, culture, or study methodology [1].

In the western region of Saudi Arabia, a prevalence of 8% was reported among adolescent school children in the age group of 13-15 years [18]. On the contrary, the current study reported that 84.9% of hospital patients seek orthodontic attention. This difference could be attributed to study setting and socio-cultural differences. In the western region of Saudi Arabia where the previous study was conducted, the population is more cosmopolitan. Whereas, Najran, in the southern region of Saudi Arabia, is very conservative with native Bedouins in the majority. Additionally, close intra-family marriages occur in Najran, which may predispose the population to various forms of genetic abnormalities [19]. The current study from Najran also reported a slight female preponderance, whereas, the study from Jeddah reported a male preponderance. It is opined that different study populations could also explain this variance as Jeddah is more cosmopolitan than Najran. 

Bimaxillary proclination was reported to be associated with various underlying skeletal patterns, facial profiles, and dental and molar relationships [3]. The majority of bimaxillary cases in the current study have convex facial profiles and Class I skeletal/molar relationships, which is similar to other reported studies [3, 18]. In Saudi Arabia and Najran specifically, most studies have reported a higher incidence of skeletal Class I relationships in the population, and this could explain the majority of the bimaxillary proclination in the current study having Class I relationships [20-22]. 

In the treatment of bimaxillary proclination, management protocols can be distributed into camouflage treatment or orthognathic surgery [23]. The principal objective of the treatment of bimaxillary protrusion is to decrease maxillary and mandibular incisal inclinations by extracting the premolars [1]. This is done to improve dentofacial esthetics and smile. Although extraction in orthodontic treatment is still an area of debate, it is still a fundamental consideration in some cases such as bimaxillary proclination. The premolars are the teeth of choice in extraction cases because they give long-term stability [24]. In the current study, premolar extraction was offered to the patients, which is in tandem with the literature. The limitation of the current analysis is that it was a hospital-based study and may not reflect what prevails in the Najran population. However, the data generated will serve as a basis for comparison with future population-based studies.

## Conclusions

Bimaxillary proclination is a common orthodontic health issue in the Najran region as observed by the high prevalence in the current study. The current study reported that 84.9% of hospital patients seek orthodontic attention. Higher occurrence was observed among females. Most of the patients presented with a Class I Angle’s relationship. All the patients were offered extraction as a treatment modality. The majority of the teeth extracted were the premolars because they give long-term stability. None of the patients went through orthognathic surgery for the correction of their bimaxillary proclination.
